# Supplementary material for: A multi-pulse ultrasound technique for imaging of thick-shelled microbubbles demonstrated in vitro and in vivo
Source: PLoS One. 2022 Nov 3;17(11):e0276292. doi: 10.1371/journal.pone.0276292 (PMC9632906; doi:10.1371/journal.pone.0276292)
Supplement: S1 File — (DOCX) [file pone.0276292.s001.docx]

S1 File

Supplementary data from: A multi-pulse ultrasound technique for imaging of thick-shelled microbubbles demonstrated in vitro and in vivo


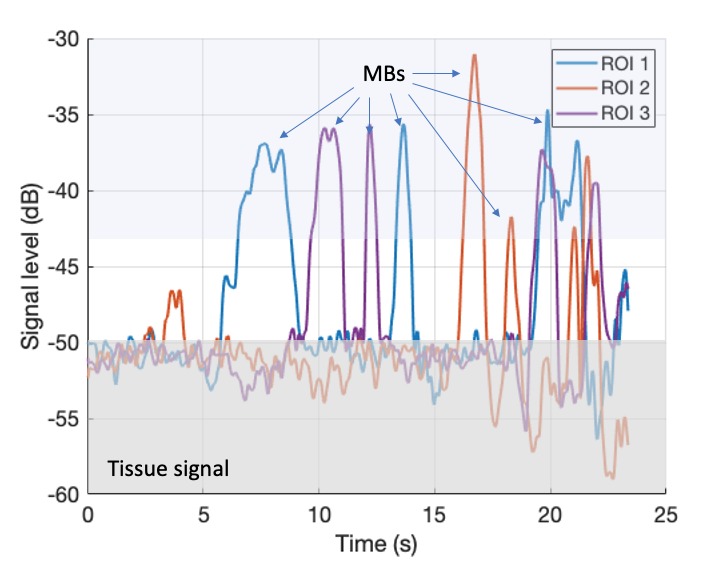


Figure S1: Example of traces retrieved from the Q-analysis tool in the GE EchoPAC software, showing how the signal from the pixels within circular ROIs of 0.5 mm diameter suddenly increased when a MB appeared within the ROI. The tissue signal is generally below -50 dB and peaks above -43 dB are considered to be from MBs.


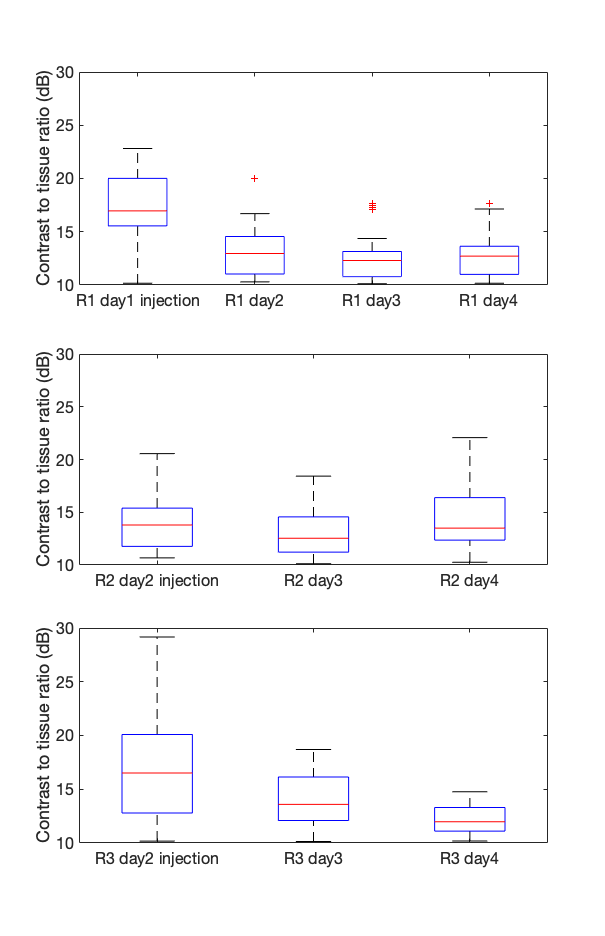


Figure S2: Summary of contrast to tissue ratio of the multi-pulse technique, when imaging thick-shelled microbubbles just after injection and after 1, 2 and 3 days. The upper panel is from Rabbit 1, the middle from Rabbit 2 and the lower from Rabbit 3. In the images recorded just after MB injection, the MBs were moving in both large and small vessels, whereas in the recordings 1 to 3 days after injection the MBs were stationary. In both cases the transducer was handheld, and the area of interest was imaged by moving the transducer laterally. Hence MBs appeared in the ROI for brief periods as can be seen from Figure S1.
