# Supplementary material for: A multi-pulse ultrasound technique for imaging of thick-shelled microbubbles demonstrated in vitro and in vivo
Source: PLoS One. 2022 Nov 3;17(11):e0276292. doi: 10.1371/journal.pone.0276292 (PMC9632906; doi:10.1371/journal.pone.0276292)
Supplement: Attachment — Submitted filename: Respons to Reviewers_2.review.pdf [file pone.0276292.s003.pdf]

We thank for the second round of thorough review done by all three reviewers. We have addressed all the comments and answered the remarks in this document. Changes made in the manuscript are commented with reference to line numbers in the revised version of the manuscript with track changes.

All answers and comments made by the authors of the paper are all in red letters in this document.

PONE-D-21-28949R1
A multi-pulse ultrasound technique for imaging of thick-shelled microbubbles demonstrated in vitro and in vivo

Thank you for submitting your manuscript to PLOS ONE. After careful consideration, we feel that it has merit but does not fully meet PLOS ONE's publication criteria as it currently stands. Therefore, we invite you to submit a revised version of the manuscript that addresses the points raised during the review process.

Please submit your revised manuscript by Jun 16 2022 11:59PM. If you will need more time than this to complete your revisions, please reply to this message or contact the journal office at plosone@plos.org. When you're ready to submit your revision, log on to https://www.editorialmanager.com/pone/ and select the 'Submissions Needing Revision' folder to locate your manuscript file.

Please include the following items when submitting your revised manuscript:

- A rebuttal letter that responds to each point raised by the academic editor and reviewer(s). You should upload this letter as a separate file labeled 'Response to Reviewers'.
- A marked-up copy of your manuscript that highlights changes made to the original version. You should upload this as a separate file labeled 'Revised Manuscript with Track Changes'.
- An unmarked version of your revised paper without tracked changes. You should upload this as a separate file labeled 'Manuscript'.

If you would like to make changes to your financial disclosure, please include your updated statement in your cover letter. Guidelines for resubmitting your figure files are available below the reviewer comments at the end of this letter.

If applicable, we recommend that you deposit your laboratory protocols in protocols.io to enhance the reproducibility of your results. Protocols.io assigns your protocol its own identifier (DOI) so that it can be cited independently in the future. For instructions see: https://journals.plos.org/plosone/s/submission-guidelines#loc-laboratory-protocols. Additionally, PLOS ONE offers an option for publishing peer-reviewed Lab Protocol articles, which describe protocols hosted on protocols.io. Read more information on sharing protocols at https://plos.org/protocols?utm_medium=editorial-email&utm_source=authorletters&utm_campaign=protocols.

We look forward to receiving your revised manuscript.

Kind regards,

Guy Cloutier, Ph.D.
Academic Editor
PLOS ONE

Additional Editor Comments:

Unfortunately, one reviewer still believes that another round of revision is necessary. He is insisting on the use of amplitude modulation in new sets of experiments for comparison. I will wait for your new responses and changes before finalizing the decision.

Sincerely,

Dr Cloutier
Academic Editor

Reviewer #1:

The authors do an adequate job at replying to the reviewer's commentary. I will just clarify one thing: The authors state that AM relies on nonlinear behaviour and their proposed method relies on radiation force and is thus mechanistically different. Typically, when we refer to nonlinear bubble vibrations we are referring to their nonlinear content (e.g. subH), and I agree that in this context (thin versus thick shell), the stated difference above is reasonable since thick-shelled bubbles don't vibrate very easily. However, the power of AM comes from the 'nonlinear' signal remaining in the fundamental band due, not specifically to nonlinear vibrations, but to a non-proportional response to two input pressures (at the fundamental - i.e. linear band). This is due to the fact that a given bubble may not vibrate at all at one pressure amplitude A, and then vibrate with a very large amplitude at a transmit pressure 2A - more than just a proportional amount. This is not a nonlinear oscillation issue, it's a lack/presence of microbubble issue. In this sense, the proposed technique works very, very similarly (if not identically) to AM, as was mentioned by both reviewers.

A nonlinear system is a system where the output is not proportional to the input. In the frequency domain, the output may be characterized by regular harmonic components (2nd, 3rd, etc.), a nonlinear fundamental component (which typically is utilized in AM), subharmonics or inter harmonics (not an integer multiple of the input frequency). A system which results in no (or little) output at a given input amplitude and some (or large) output at twice the input amplitude is a typical example of a nonlinear system.

Our method is fundamentally different from AM which now also is demonstrated by in vitro experiments, since we now have included in vitro AM imaging of the thick-shelled MB. The

images are included in figure 4, and commented in the text in lines 127 – 132, 259 – 265 and 470 – 477 in the manuscript with tracked changes.

Reviewer #2:

Thank you for addressing most of my comments.

- I am not certain I understand your response to Question 1 about additional in vitro experiments:
"Unfortunately, we do not have the needed infrastructure for conducting such experiments at this point."
What infrastructure are you referring to? Targeting MB in vitro is not that difficult. For example you can look at PMID: 19411212.

We are aware that such experiments have been done earlier and see that it could contribute to the understanding of how bound microbubbles respond to ultrasound. However, setting up such an experiment for the first time would require significant resources, both in terms of time spent and materials and equipment needed. This is not something which is possible for us to do at this time, but we will keep it in mind for future projects.

- I also think that you could do a better job at describing and quantifying your CTR in vivo results L351-356. Since you have 172 + 205 MB you have analyzed at different time points, it would be interesting to describe these results in more detail using a figure.

Figures showing the CTR-levels are added as supplementary information, and are referred to in the text, line 370 – 372 in the manuscript with tracked changes.

There are still a few typos that may require your attention
L252 : Grammar : satisfactory is an adjective. In this phrasing your should use an adverb such as "adequately" ? or perhaps write : "was low, as expected" - corrected
L313: Grammar : ... in Table 1. "Additionally to the results in Table 1," RF data from images... - corrected
L319 : In table1 : I believe that a period is missing for MI=0.7 3HP standard deviation - corrected
L343: Please add : 16.8dB "for flowing MB" - corrected
L353 : Please add : 13.3 dB "for static MB" - corrected
L378 : you say 30 dB but the color bar is suggesting 35 dB. Please verify. – sorry for the typo. It was actually 36 dB
L383 : Is is common to display DAPI in blue. I understand this is pseudo colouring but it is confusing when looking at the image. If possible please use blue for DAPI. – in principle it is possible to change the DAPI coloring to blue, but we find that the contrast to the black background is better with green in these images. Hope the reviewer and editor is OK with that.
L516 : Grammar : please add a period and cut in two sentences : ... [14,15]. This is also

supported by... - corrected
L518 : replace "towards" with "of" - corrected

Reviewer #3:

I would like to thank the author for revising the manuscript. However, I still believe it would be worthwhile to properly investigate the advantage of the manipulation pulse both in-vitro and in-vivo. While the modulation has theoretical advantages over power Doppler, this is not quite demonstrated in the manuscript. I also think it would be worthwhile to compare the proposed sequence to Amplitude modulation in a flow phantom setup (as done for PI).

We believe that the in vivo data shown in the results of the study show that the MBs with thick shell are possible to image both when they are in blood flow and when they are stationary, and likely bound. Additional in vivo experiments will be very time consuming and costly, and not possible to add at this stage for the current publication. We have now added in vitro data where the novel imaging method is compared to both PI and AM, and show that in a controlled lab environment, the MBs are not possible to image with either PI or AM with sufficient tissue suppression. Hence, we have a sound reason for not including those methods in the animal experiments.

Amplitude modulation was not (and still is not) available on the GE Vivid E9 system which was used in the original data collection for this publication. We have however now gotten access to a GE Vivid E95, where also Amplitude modulation is implemented, and could include tissue mimicking phantom imaging in the results, as requested by the reviewer. The resulting images are included in a new version of Figure 4 and commented in the manuscript in lines 127 – 132, 259 – 265 and 470 – 477 in the manuscript with tracked changes.

(l. 326) “However, due to motion, and flashing artefacts when using the intermediate manipulation pulses in vivo, the in vivo results were recorded without manipulation pulses at MI=1.”
Comment: The addition of the manipulation pulse will decrease the pulse repetition frequency between the imaging pulses, but should also increase the bubble displacement and improve detection from background tissue. Similar to Power Doppler, the detectability of the microbubbles will be tied to the amount of displacement detected relative to surrounding tissue displacement.

The decreased PRF might be the main cause of the flashing artefacts from the tissue that was seen in the image when manipulation pulses were included. The implementation of the method was not optimal due to limited access to all transmit and receive settings. With the implementation that was possible at the time of recording in vivo images, our conclusion was that the best image quality in vivo was achieved by dropping the intermediate pulses, since the MBs could be clearly detection both during flow and when they were static even

without intermediate pulses. The detectability of the microbubbles is not strictly tied to the amount of displacement because there can also be amplitude effects (e.g., due to changes in the shell resulting in variable backscattering).

(From reviewer response) "Gas vesicles typically have very thin protein shells (approx. 2nm or less) and the vesicle itself has a cylindrical dimension with diam of 50-100 nm and length of 100-1000 nm. The PVA bubble has a thick polymer shell of approx. 200 nm. Our GE Vivid scanner did not have an implementation of AM and there is no reason to believe that AM will work adequately for thick-shelled PVA bubbles. The buckling-effect and compression-only behavior for example seen with SonoVue (having a lipid shell of approx. 2 nm) is unlikely with a thick polymer shell of approx. 200 nm."
Comment: While it is true that gas vesicle differs from thick shell microbubbles, there is experimental and theoretical evidence in literature that thick shell microbubbles will buckle through reversible collapse. In particular see [P. Marmottant et al. Buckling resistance of solid shell bubbles under ultrasound, JASA, 129 (3), 2011]. As the CTR achieved by AM is strongly affected by the buckling dynamics of bubbles, there is reason to believe that AM will perform much better than PI [ref: C. Tremblay-Darveau, IEEE Trans UFFC, 65 (8), 2018]. It seem highly relevant, and the authors should ideally perform an in-vitro validation of their technique to AM (which should be readily available on most clinical systems) even if done on a different clinical system.

An in vitro test with AM has been performed and included in the paper. See figure 4 and changes in the manuscript, lines: 127 – 132, 259 – 265 and 470 – 477. Shell buckling is mentioned as a possible reason for AM signal, but as is shown in Fig 4, a high MI is needed also for AM to be able to detect the MBs, hence the tissue suppression is not adequate for imaging in vivo. We have also included references to Tremblay-Darveau et al. and Marmottant.

(From reviewer response) " One might argue that the technique has similarities with conventional Doppler but Doppler techniques are made for imaging an existing flow, not to induce a translation due to ultrasound radiation force as with our method. That is why we want to use imaging pulses tailored to the organ size and depth of interest and intermediate manipulation pulses that are tailored to maximize the radiation force on the PVA bubbles. Also, Doppler makes use of transmit pulses consisting of several pulse oscillations (resulting in reduced range resolution) and several pulse packets needs to be transmitted in each beam direction for spectral analysis (to construct an adequate clutter filter for suppressing strong, slow moving scatterers and to estimate blood velocities)."
Comment: I agree that pulses used for CW and PW are typically quite long, but Colour or Power Doppler pulses are typically of length comparable to that of Contrast pulses. If the manipulation pulse is taken out (as done in the in-vivo section), then the pulse sequence is equivalent to a N=2 ensemble power Doppler sequence (and is not novel). It would be worthwhile to try the power Doppler mode on the GE machine (using the lowest PRF) and see how it compares to the proposed pulse sequence. It is also not clear to me why the manipulation pulse is taken out for the in-vivo imaging. The authors claim that using the

manipulation pulse causes too much tissue motion clutter (l.326), but clutter is a fundamental limitation of any Doppler system. Does this mean that the implementation of the manipulation pulse is not practical in-vivo? I understand the theoretical advantages of using a long burst for the manipulation pulse, but this is not demonstrated in-vivo in the manuscript.

We agree that the method has similarities with power Doppler. However, conventional power doppler implementation is not suitable for detecting thick shelled MBs. We have tested Power Doppler on GE Vivid E9 with the 11L transducer in the tissue mimicking phantom, and imaged stationary MBs (after the pump had stopped). We tested various combinations of parameters (available clinical settings) and MI up to 1.2. The conclusion was that the MB detection in combination with tissue suppression was not possible to achieve with any of the setting combinations. Since this method is generally not considered to be a contrast enhancement method, we have not included data on this in the paper, but rather focused on the comparison to conventional contrast sequences such as PI and AM.

We have demonstrated the usefulness of the manipulation pulses in in vitro tests. In vivo tests showed reduced robustness in the presence of tissue motion (increased clutter and flashing artefacts) due to suboptimal implementation. The implementation of the novel method was done by changing the transmit and receive sequence of already existing imaging applications, hence the overall signal processing chain was not fully optimized at the time of the experimental work. A modified detection sequence based on power Doppler using short (e.g., 1-cycle) pulses of suitable ensemble length, for optimal spatial resolution and clutter/tissue filtering, with intermediate manipulations pulses of suitable frequency and duration, for inducing bubble decorrelation, is probably the optimal implementation of this method. This has been described in future work, line 518 – 524.

(From reviewer response) "The aim of the current paper was not to describe various contrast pulse sequences and reference 3 provides a good overview of such sequences" Comment: The reader should be able to infer from the introduction where the authors work sits within the existing literature. For instance, is there any literature work describing how PI/AM/AMPI do not work well with hard shell microbubble? Any theorical work on solid shell microbubble that could be use to justify the pulse sequence (Marmottant's paper on stiff shell buckling dynamic seems relevant)? If the goal of the modulation pulse is to displace microbubble, some citation of papers describing acoustic radiation forces on bubbles is also relevant.

A couple of sentences have been added in the introduction about radiation force related to contrast enhanced imaging and the following references has been added, see line 48 – 52 in the manuscript:

14. Patil, A.V., et al., *Real-time technique for improving molecular imaging and guiding drug delivery in large blood vessels: in vitro and ex vivo results.* Molecular imaging, 2011. **10**(4): p. 7290.2011. 00002.

15. Zhao, S., et al., *Radiation-force assisted targeting facilitates ultrasonic molecular imaging.* Molecular imaging, 2004. **3**(3): p. 15353500200404115.

16. Lum, A.F., et al., *Ultrasound radiation force enables targeted deposition of model drug carriers loaded on microbubbles.* Journal of controlled release, 2006. **111**(1): p. 128-134

Reference to the Marmottant paper has been added in the discussion. Line 474.

(From reviewer response) " (p.19 l.437) 'We have not been able to verify the presence of such effects in the current work'. -> That being said, it is unlikely that the acoustic pulses moved the bounded bubbles in the in-vivo experiment. -> We agree. Knowing exactly what the cause of the detection signal from each MB in an in-vivo situation is impossible (except for maybe in intra-vital microscopy at very high magnification and frame rate). In vitro, it is possible to study single bubble behavior, as has been done in Kohtapalli at al (2015)." Comment: This limitation should be described in the Discussion section, including possible hypothesis from existing literature.

We have already discussed both the MB translation due to radiation force and the possibility of shell perturbation, partial rupture or deflation, and refer to the very relevant paper by Kothapalli where the same kind of PVA MBs were studied.

It is also possible that we induce motion with bound bubbles, resulting in detachment after some time. In most situation, a component of the radiation force will act in the flow direction of the capillary. We noticed that the detected bubbles would have a tendency to disappear after being imaged for a while (time of disappearance varying from about 5 seconds to a few minutes). A sentence regarding this has been added in line 543 – 544 in the manuscript with tracked changes.
