# Supplementary material for: A multi-pulse ultrasound technique for imaging of thick-shelled microbubbles demonstrated in vitro and in vivo
Source: PLoS One. 2022 Nov 3;17(11):e0276292. doi: 10.1371/journal.pone.0276292 (PMC9632906; doi:10.1371/journal.pone.0276292)
Supplement: Attachment — Submitted filename: Response to editor.pdf [file pone.0276292.s004.pdf]

Trondheim, Sept. 07th 2022

Response to editor regarding the manuscript "A multi-pulse ultrasound technique for imaging of thick-shelled microbubbles demonstrated in vitro and in vivo" PONE-D-21-28949R2.

Editors comment:
To comply with the PLOS data policy, please ensure data underlying all of your findings are made available, outlining in your data availability statement how the data can be accessed. Alternatively, if it is not possible to make all data available, please remove the parts of the manuscript for which the raw supporting data cannot be provided and include a detailed justification in your response-to-reviewers that outlines which data were removed and if/how your conclusions are affected. We will not be able to proceed further with your manuscript until these concerns are addressed.

Response to editor
We have been in contact with representatives from GE Healthcare and they have given us permission to share raw IQ data from the in vitro recordings which form the basis of the results presented in Table 1 and Figure 5.
IQ data, including a time axis and demodulation frequency is included in the data repository as .mat-files.
We have made changes to the "Data Availability" statement and the comment following the statement. It is no stated that "Yes" all underlying data is made available, and the comment is as follows:
Underlying data is fully available at the following repository:
https://doi.org/10.11582/2021.00072

We do not see any need for making any changes to the manuscript submitted in June 2022. However new files have been uploaded, where the "Manuscript with Track Changes" is the same as the "Manuscript". The files are named "Revised_manuscript_3.docx".

Best regards,
Sigrid Berg
