# Supplementary material for: A multi-pulse ultrasound technique for imaging of thick-shelled microbubbles demonstrated in vitro and in vivo
Source: PLoS One. 2022 Nov 3;17(11):e0276292. doi: 10.1371/journal.pone.0276292 (PMC9632906; doi:10.1371/journal.pone.0276292)
Supplement: Attachment — Submitted filename: Response to reviewers.docx [file pone.0276292.s002.docx]

We thank for the thorough review done by all three reviewers. We have addressed all the comments and answered the remarks in this document. Changes made in the manuscript are commented with reference to line numbers in the revised version of the manuscript.

All answers and comments made by the authors of the paper are all in red letters in this document.

PLOS ONE

Journal Requirements:

When submitting your revision, we need you to address these additional requirements.

1. Please ensure that your manuscript meets PLOS ONE's style requirements, including those for file naming. The PLOS ONE style templates can be found at 
<https://journals.plos.org/plosone/s/file?id=wjVg/PLOSOne_formatting_sample_main_body.pdf> and 
<https://journals.plos.org/plosone/s/file?id=ba62/PLOSOne_formatting_sample_title_authors_affiliations.pdf>

2. Thank you for stating the following financial disclosure:

SB: Liaison Committee between the Central Norway Regional Health Authority (RHA) and the Norwegian University of Science and Technology (NTNU) 
SB and RH: Research Council of Norway (240410/F20) 
SB, SE, LD, KC and RH: European commission (7th framework program, 3MiCRON (245572) project)
 
Please state what role the funders took in the study.  If the funders had no role, please state: "The funders had no role in study design, data collection and analysis, decision to publish, or preparation of the manuscript." 
If this statement is not correct you must amend it as needed. 
Please include this amended Role of Funder statement in your cover letter; we will change the online submission form on your behalf.

3. Thank you for stating the following in the Competing Interests section:

 SB and RH are co-inventors of a patent describing the ultrasound contrast imaging method (EP3125770). LD is the founder of Surflay Nanotec GmbH, the company that produced the microbubbles used in this work.

Please confirm that this does not alter your adherence to all PLOS ONE policies on sharing data and materials, by including the following statement: "This does not alter our adherence to  PLOS ONE policies on sharing data and materials.” (as detailed online in our guide for authors <http://journals.plos.org/plosone/s/competing-interests>).  If there are restrictions on sharing of data and/or materials, please state these. Please note that we cannot proceed with consideration of your article until this information has been declared.

Please include your updated Competing Interests statement in your cover letter; we will change the online submission form on your behalf.

4. In your Data Availability statement, you have not specified where the minimal data set underlying the results described in your manuscript can be found. PLOS defines a study's minimal data set as the underlying data used to reach the conclusions drawn in the manuscript and any additional data required to replicate the reported study findings in their entirety. All PLOS journals require that the minimal data set be made fully available. For more information about our data policy, please see <http://journals.plos.org/plosone/s/data-availability>.

Upon re-submitting your revised manuscript, please upload your study’s minimal underlying data set as either Supporting Information files or to a stable, public repository and include the relevant URLs, DOIs, or accession numbers within your revised cover letter. For a list of acceptable repositories, please see <http://journals.plos.org/plosone/s/data-availability#loc-recommended-repositories>. Any potentially identifying patient information must be fully anonymized.

Important: If there are ethical or legal restrictions to sharing your data publicly, please explain these restrictions in detail. Please see our guidelines for more information on what we consider unacceptable restrictions to publicly sharing data: <http://journals.plos.org/plosone/s/data-availability#loc-unacceptable-data-access-restrictions>. Note that it is not acceptable for the authors to be the sole named individuals responsible for ensuring data access.

We will update your Data Availability statement to reflect the information you provide in your cover letter.

Additional Editor Comments:

Three expert reviewers provided detailed and relevant comments. Among many other issues, some "solvable" flaws were identified. It is clear that the framework of the comparative evaluation with different sequences is seriously questioned since the acoustic pressure used and frequencies are different. Additional experiments would be required to better support your statements and quantitative analysis would also be required in vivo.

We have explained in the comments to the reviewers that the main purpose of this paper was not to compare methods, since the commercially methods which works nicely for thin-shelled MBs, clearly does not work for thick-shelled MBs. We have made some new recordings, which are replacing the pulse inversion images in figure 4. In the new recordings, the gain has been adjusted so that the level of electronic noise from the inside the flow tube is the same at all three pressures. As can be seen, the MBs are visible at high MIs also for pulse inversion, but tissue suppression is no longer possible. In addition we have added more information on how the CTR is calculated in-vivo, and the exported traces (txt-files) from the Q-analysis method in the software EchoPAC is added as supplementary data in the repository.

[Note: HTML markup is below. Please do not edit.]

Reviewers' comments:

Reviewer's Responses to Questions

**Comments to the Author**

1. Is the manuscript technically sound, and do the data support the conclusions?

The manuscript must describe a technically sound piece of scientific research with data that supports the conclusions. Experiments must have been conducted rigorously, with appropriate controls, replication, and sample sizes. The conclusions must be drawn appropriately based on the data presented.

Reviewer #1: Yes

Reviewer #2: Partly

Reviewer #3: Partly

2. Has the statistical analysis been performed appropriately and rigorously?

Reviewer #1: N/A

Reviewer #2: No

Reviewer #3: N/A

3. Have the authors made all data underlying the findings in their manuscript fully available?

The [PLOS Data policy](https://eur03.safelinks.protection.outlook.com/?url=http%3A%2F%2Fwww.plosone.org%2Fstatic%2Fpolicies.action%23sharing&data=04%7C01%7Csigrid.berg%40sintef.no%7Cbc069de421ab4e71b8d708d9e6718737%7Ce1f00f39604145b0b309e0210d8b32af%7C1%7C0%7C637794198994625074%7CUnknown%7CTWFpbGZsb3d8eyJWIjoiMC4wLjAwMDAiLCJQIjoiV2luMzIiLCJBTiI6Ik1haWwiLCJXVCI6Mn0%3D%7C3000&sdata=vrQ5zzp%2FYqUBO6vtVuOABGfzVnkOeiAWJEb1iYXpfgs%3D&reserved=0) requires authors to make all data underlying the findings described in their manuscript fully available without restriction, with rare exception (please refer to the Data Availability Statement in the manuscript PDF file). The data should be provided as part of the manuscript or its supporting information, or deposited to a public repository. For example, in addition to summary statistics, the data points behind means, medians and variance measures should be available. If there are restrictions on publicly sharing data—e.g. participant privacy or use of data from a third party—those must be specified.

Reviewer #1: Yes

Reviewer #2: Yes

Reviewer #3: Yes

4. Is the manuscript presented in an intelligible fashion and written in standard English?

PLOS ONE does not copyedit accepted manuscripts, so the language in submitted articles must be clear, correct, and unambiguous. Any typographical or grammatical errors should be corrected at revision, so please note any specific errors here.

Reviewer #1: Yes

Reviewer #2: Yes

Reviewer #3: Yes

5. Review Comments to the Author

Please use the space provided to explain your answers to the questions above. You may also include additional comments for the author, including concerns about dual publication, research ethics, or publication ethics. (Please upload your review as an attachment if it exceeds 20,000 characters)

Reviewer #1: The authors present a succinct piece of work using a new imaging sequence that is tailored for stiff microbubbles. The new imaging sequence is very similar to amplitude modulation, in that it is measuring the difference between the response of a bubble population over time, which can be due to translation, bubble rupture, or other effects. I would have liked to see a direct comparison between the new sequence and AM, not PI as the authors did. The comparison between PI, indeed, was taken at entirely different frequencies and pressure amplitudes, and I’m not sure it’s a fair comparison. The work seemed to be conducted robustly, and in that regard the paper deserves eventual publication. I would perhaps suggest to the authors that, in addition to my comments below, the authors comment on the similarly between AM, PI and their new sequence since the physics isn’t different. This approach essentially is trying to get away from bubble vibration at all (since they don’t vibrate very well at low MI) and is trying to focus on signal ‘difference’ due to motion, etc. What is the expected frequency content of the resulting signal, would it work best on big or small bubbles, would it also work on lipid bubbles, etc.

Both PI and AM and combinations thereof (often referred to as contrast pulse sequences) rely on a bubble response that is adequately nonlinear at low incident pressure. At higher incident pressure, nonlinear wave propagation will result in limited specificity and these methods then fail to differentiate tissue and contrast echoes. All multi-pulse techniques (PI, AM, our technique) will benefit from bubble motion due to flow. However, for bubbles in capillaries, the flow is so slow that it will not contribute to any signal decorrelation between consecutive transmit pulses. Our method does not rely on a nonlinear bubble response, but the method can certainly benefit from nonlinearities (e.g., due to changes or small cracks in the thick bubble shell). The frequency content of the resulting contrast signal will be similar to the transmitted frequency band. The method works best when we can obtain a high radiation force on the bubbles, hence for bubbles with a high extinction cross-section (high attenuation) that tolerate high incident pressure amplitudes. The method can in principle work for lipidic bubbles but conventional lipidic agents with very thin shells such as SonoVue are fragile and probably not well suited for this method.

Since the receive frequency is the double of the transmit when using PI, the transmit is typically in the lower range of the transducer bandwidth. In the clinical scanner and transducer (GE E9 with 9L transducer) that was available to us, PI is only available with transmit frequency at 3 MHz and 3.6 MHz. Cardiac transducers typically also have a PI-applications implementation available, but that is on even lower transmit frequencies. The 11L transducer did not have a PI application available, but we found that the implementation of the novel method worked better on the 11L, and therefore continued to use that for both in vitro and in vivo experiments. The 11L allows for higher transmit frequencies than the 9L.

Line 30: “In the later years […] I might suggest adding a reference here (like a review article or something, perhaps a Klibanov one?)

The following references has been added:

6.	Lentacker, I., et al., *Design and evaluation of doxorubicin-containing microbubbles for ultrasound-triggered doxorubicin delivery: cytotoxicity and mechanisms involved.* Molecular therapy, 2010. **18**(1): p. 101-108.

7.	Escoffre, J.-M., et al., *Doxorubicin liposome-loaded microbubbles for contrast imaging and ultrasound-triggered drug delivery.* IEEE transactions on ultrasonics, ferroelectrics, and frequency control, 2012. **60**(1): p. 78-87.

8.	Snipstad, S., et al., *Sonopermeation Enhances Uptake and Therapeutic Effect of Free and Encapsulated Cabazitaxel.* Ultrasound Med. Biol., 2021. **47**(5): p. 1319-1333.

9.	Klibanov, A.L., *Microbubble Contrast Agents: Targeted Ultrasound Imaging and Ultrasound-Assisted Drug-Delivery Applications.* Investigative Radiology, 2006. **41**(3): p. 354-362 10.1097/01.rli.0000199292.88189.0f.

10.	Un, K., et al., *Efficient suppression of murine intracellular adhesion molecule‐1 using ultrasound‐responsive and mannose‐modified lipoplexes inhibits acute hepatic inflammation.* Hepatology, 2012. **56**(1): p. 259-269.

11.	Villanueva, F.S., et al., *Microbubbles Targeted to Intercellular Adhesion Molecule-1 Bind to Activated Coronary Artery Endothelial Cells.* Circulation, 1998. **98**(1): p. 1-5.

Line 33: “less resonant”. I think you’ll need to clarify this. What is a less resonant bubble? You mean it’s resonant oscillation magnitude is smaller than that of a resonant lipid-coated bubble of the same size and shell properties? I’m not sure that’s such an easy comparison but I do see your point. Please clarify

Sentence has been rephrased to: A thick shell will in general have higher viscosity and therefore introduce higher damping of the bubble oscillation compared to a thin shell. (line 37-39)

Line 35: “Compared to ultrasound imaging of thin-shelled MBs, higher mechanical indices are typically required to drive thick-shelled MBs into nonlinear oscillations.”.
• Please provide a reference
• It’s generally understood that thick-shelled bubbles need to break to elicit sufficient nonlinear oscillations (i.e. destructive nonlinear behaviour). Is this what the authors are referring to?

As stated above nonlinear signal can arise from changes or small cracks in the shell, but if there is some flexibility in the shell oscillations can also be achieved at higher MIs.

The following reference has been added:

12.	Hoff, L., P.C. Sontum, and J.M. Hovem, *Oscillations of polymeric microbubbles: Effect of the encapsulating shell.* The Journal of the Acoustical Society of America, 2000. **107**(4): p. 2272-2280.

Line 38: “[…] while supressing harmonic components from soft tissues.” These pulses are also designed to supress fundamental signal from soft tissues, too. Please modify sentence to account for this.

Sentence has been rephrased to: Contrast harmonic imaging methods typically rely on transmit pulses with very low mechanical indices, sufficient to invoke nonlinear scattering from thin-shelled MBs while suppressing harmonic components, as well as the strong fundamental component, from soft tissue. (line 41-43)

Microbubble preparation: Were these measured by Coulter Counter also? Microscopy methods will not capture bubbles below 1 um (biased result). How confident are the authors in this size distribution (Fig 1)? A concentration of 1e8 is quite low, which might be a symptom of missing the smaller bubbles.

In line 102 there was a typo, the original stock concentration of MBs were given by the partner producing the MBs as 1e9 MB/ml, measured by counting MBs in a Neubauer cell under a laser scanning microscope. This information has been added in line 102-103. This concentration is the same as given in line 123, where we specify that the stock solution is diluted to 2e8 MB/ml before injection into animals.

To get a sense of the size distribution, the MBs were imaged with a microscope, and their size distribution was measured based on the images. We agree that very small bubbles could be “lost” in the imaging process, due to the image resolution. However, as can be seen from the plot in the right of Figure 1, the fraction of MBs at 2 micron is only around 10%, so it is not expected a large amount of very small bubbles.

In-vitro testing: Clearly the comparison made between the novel pulse sequence and traditional pulse-inversion isn’t valid, since the MI’s are not the same and neither are the frequencies! I’m a little confused: If the authors used PI at an MI equal to that of their new sequence (MI=1), they surely would obtain usable contrast-images. Is this true? For example, the ‘new method, 2 pulses’ is actually sort of similar to AM (since there is no intervening pulses), but taken at MI=1.
In fact, this novel scheme works essentially as amplitude modulation. The idea is that the intervening pulses either move or distort the bubbles (but not tissue), and then upon subtraction of two pulses, you are left with ‘nonlinear fundamental’ signal – that is signal at the fundamental (which is linear) due to the presence or absence of a target. I’d like to see how this new sequence compares with traditional AM (which is almost the same as ‘new method, 2 pulses’).

PI is shown at MI of 0.1 to 0.6. Usually PI is used in the very low MI range (0.03 to 0.2), where the method typically will result in images with nice contrast to tissue ratio when imaging thin-shelled bubbles. We show that low MI does not provide adequate detection of thick-shelled-bubbles, and when increasing the MI to levels where there is some signal from the MBs, the tissue signals is very high (as expected for a harmonic method such as PI). The new method, on the other hand, works well at higher frequencies and at higher pressures, where both bubble detection and tissue suppression are adequate. It is possible to produce a nice image of the bubbles using PI at high MI in the flow-phantom where we have a black background due to zero scattering from the water-filled channel. However, in a clinical situation we are typically interested in detecting bubbles in the micro-circulation and not in large arteries or veins. It is then necessary to suppress the strong tissue signal and harmonic methods then fail at high MIs due to nonlinear wave propagation from the tissue.

To better illustrate the results from PI, a new recording with gain adjusted so that the background signal from the flow tube was similar at all MI settings. It turns out that the clinical scanner automatically compensates for low MI by increasing the (analog?) gain, hence the tissue signal appears brighter than it should at low MI compared to high MI.

The mechanisms of action are essentially different with AM and our method. Our method relies on a physical translation of the bubble due to a radiation force and potentially also alterations in the back-scattering due to small cracks or other changes in the thick PVA shell. From in vitro experiments, the effect of physical translation seems to be the dominant effect for these PVA bubbles.

AM does not rely on radiation force but on a nonlinear response. AM has been shown to work especially well on some agents such as SonoVue where you get a compression-only behavior with buckling of the shell at very low incident pressures. As the amplitude is increased (typically by a factor of 2) the bubble responds with a more conventional compression-expansion behavior and the result of the AM-sequence is especially a nonlinear fundamental component. SonoVue has a very thin lipidic shell (approx. 2 nm) and it is highly unlikely that the thick PVA shell (approx. 200 nm) of our bubble will respond in a similar way with buckling at low incident pressure.

Reviewer #2: In this manuscript entitled “A multi-pulse ultrasound technique for imaging of thick
shelled microbubbles demonstrated in vitro and in vivo”, the authors report a multi-pulse subtraction imaging technique, with interleaved manipulation pulses, to image thick shelled polymer microbubbles in vitro in a flow loop and in vivo in a rabbit model of inflammation. Their in vitro experiments are conducted using very low concentration of MB in a commercially available flow phantom. Their in vitro results indicate that their non-destructive subtraction imaging scheme, which, on responding microbubbles, can result in a CTR of 23dB, which is good. This is attributed to a translation of the MB of up ~4um, which is roughly the diameter of one MB. The manipulating pulses can theoretically be modified (pressure, length, frequency) or even completely removed, which they did for their in vivo experiments.

In vivo, they used a modified version of their imaging approach (without manipulation pulse) for the molecular imaging of ICAM1-targeted MB using and anti-ICAM1 decorated MB using biotin streptavidin conjugation, which is an adequate conjugation scheme for a proof of principle study. They demonstrate that MB can be detected at 24h when MB are injected at 2h post injury and 72h when injected at 24h post injury but not with control MB. This is supported by histological fluorescence imaging in N=1 rabbit per condition.

Overall, this reports contains some interesting data but (1) suffers from unsupported statements and inadequate experiments in vitro and (2) lacks adequate quantitative analysis in vivo (essentially N=1 and non-analysis of longitudinal data)

MAJOR FLAWS
1- For instance, since their imaging application is targeted MB, it is not acceptable to only characterize MB in flowing conditions in vitro. Especially since their approach relies on MB translation caused by radiation force. Indeed, one has to wonder if that translation will be hindered once MBs are attached. In vitro experiments with targeted MB should be performed. How many MB will respond ? Will they roll with successive pulses? Or detach ?

In vitro experiments show that conventional pulse inversion (PI) fails to adequately image free, untargeted PVA bubbles in a tissue-mimicking phantom and PI would therefore most likely also fail to image the PVA bubbles when targeted. Our method is thus of interest for imaging of the PVA bubbles (and potentially other thick-shelled bubbles) in general (targeted or not). In vitro tests were performed on untargeted PVA bubbles whereas in vivo results indicate that both targeted and untargeted PVA bubbles can be detected.

In-vitro experiments with targeted MBs and cells in a flow environment would be interesting. Unfortunately, we do not have the needed infrastructure for conducting such experiments at this point. From the in-vitro images we see that we can image single MBs when the flow pump has stopped. Under these conditions the MBs can move in the direction of the ultrasound beam, but do not move perpendicular to the beam. From the in-vivo imaging, we see that MBs are possible to image even when they are stationary, and possibly within small vessels. We cannot be sure of the vessel size, or by what mechanism the MBs have become stationary in the US image, but the results indicate that the imaging method is not depending on free flowing MBs.

We agree that targeting may hinder or diminish the translation effect due to radiation force and it will probably also reduce the fraction of MBs being detected. During imaging we observed that the detected PVA-bubbles could disappear after being imaged for some time (typically ranging from about 5 seconds to several minutes) potentially indicating some sort of rolling and eventual detachment. A comment about this has been added in line 532-537.

2- Also, in the first part of the manuscript, the authors optimize the manipulation pulses in vitro but end up using a different imaging scheme in vivo without manipulation pulses. It would be relevant to repeat the in vitro experiments with the conditions used in vivo (no manipulation) to allow comparison and understand the importance of the manipulation pulses.

In the bottom left of Figure 4, and example image of the novel method without manipulation pulses at MI=1 is shown. In the analysis presented in Figure 5 and Table 1, data from recordings without manipulation pulses was not included originally, however we have data recorded at 8 MHz and MI=1 with 3 and 5 half periods without manipulation pulses. This is now included in the text of revised manuscript. (line 326-329)

3- Subtraction methods are known to be sensitive to motion. Yet their images (cf Fig 7) clearly do not reflect this reality, which is clearly visible in the in vivo supplementary videos. This limitation should be disclosed and discussed. Why is there so much movement artifact with rabbit 3 ? How does it affect the CTR ?

We agree that motion artifacts are a problem in the proof-of-concept implementation presented in this paper. In Fig. 7 we have chosen frames where the tissue is not flashing into view, so that the maximal CTR can be shown. We have added a comment in the discussion about the motion artifacts seen in the supplementary videos, line 499 to 500.

4- Why are only a fraction of MB responding to this imaging scheme (Figure 5)? Please quantify that. The reviewer wonders if it is fair to quantify only responding MB in table 1. Since you report results on 6-10 MB in table 1, how did you choose which MBs to analyze ? There are clearly more than 10 MB in Fig4. How did you account for the MBs that do not give signal with your method (cf Fig 5 A and B)?

It is common that not all bubbles respond equally to a contrast pulse sequence. This is also the case when for example using PI and SonoVue. As the mechanical index is increased more bubbles will become visible (while others are destroyed). For harmonic techniques the main problem at higher MIs, in addition to bubble destruction, is that specificity is sacrificed due to nonlinear wave propagation.

The MBs that were chosen for analysis were those that responded best to the ultrasound in order to display the optimal CTR that we can achieve with the current settings. When comparing the upper left and upper right image in Fig 5, it is apparent that not all MBs are imaged in the contrast mode, however those who are sensitive to the method have a very high CTR. We have specified in the abstract and the results, that the CTR is “up to 23 dB”. We have also added the word “brightest” in line 324 to describe the selection of MBs.

We believe that manipulation pulses with even higher MIs or more half cycles would increase the fraction of detected MBs. With the clinical system used in this work, it was not possible to increase the pressure or include manipulation pulses with a different burst length than the imaging pulses. This is commented in the discussion – line 492.

5- I have never seen PI harmonic imaging with such poor tissue cancellation performance (cf Figure 4). Was this contrast package the commercial package from GE or was it home-made ? Please specify in the methods. Also, an additional in vitro image with a lipid MB, perhaps in supplementary data, would be appropriate to convince the readers of the proper coding of the PI sequence.

The images the upper part of Figure 4 in the original manuscript were recorded by the clinical contrast application on the GE Vivid E9 system and with the 9L transducer. It seems that the scanner automatic adjusts the gain depending on the chosen MI setting, so even though the user does not change the gain setting on the scanner, the level of electronic noise that shows in the images varies with power (transmitted pressure/MI). This is particularly visible in the flow tube, where it can be seen that the grayscale level is much higher in the image recorded with MI=0.1 than the one recorded with MI=0.4. We therefore recorded some new images, where the gain was adjusted so that the level of electronic noise within the flow tube was similar in all images. (Text added in line 265-267.) The overall result is still the same. At low MI the tissue signal is low, but the thick-shelled MBs are not possible to detect, whereas at high MI, the signal from MBs is just as high as the signal from the tissue.

A screenshot from a recording of SonoVue recorded with the same equipment, and MI of 0.08 is shown in the left side in the images below. The concentration of SonoVue is significantly higher in this example than in the images of Figure 4. The tissue signal is however similar in the SonoVue example and the new recording in the upper left corner of Figure 4, where the MI is 0.1 (also shown to the right below for comparison):

6- Moreover, I strongly encourage the authors to use and report the same dynamic range in all images. This is an important parameter that largely affects the visual rendering of CTR

We agree that dynamic range is important. Information about dynamic range has been added in the figure captions.

7- Although this is a proof of principle study, reporting data with N=1 is never a good approach. Perhaps one way to bring more robustness to the study would be to analyze the images longitudinally at different time points (48h vs 72h), which you state you have performed in the methods, but are not reporting or quantifying. For Rabbit 1, you could even do 24h, 48h and 72h. L461: replace “days” by “48 hours in one animal”.

The analysis of CTR has been specified more precisely in the manuscript: In the analysis, images from all timepoints after MB injection has been included (Rabbit1: t = 0, 24, 48 and 72h after injection, Rabbit 2 and 3: t = 0, 24 and 48h after injection). And the CTR for the period just after injection was hence calculated based on t=0-images, and the CTR of stationary MBs was based on images from t=24, 48 and 72h. The largest difference in CTR was between t=0 and the other timepoints, since the amount of MBs is larger just after injection and that they are flowing in both large and small vessels. At the later timepoints, the MBs are fewer and mostly stationary and possibly attached to endothelial cells or taken up by macrophages, resulting in a lower CTR.

In the image below, a screenshot from the EchoPAC software is shown. Eight ROIs (seen as small circles in the ultrasound image on the left) in each recording can be selected. When an MB passed through the ROI a local peak will appear in the trace plot shown. The intensity of the peaks varies between MBs, and the CTR is calculated based on the mean of all local peaks above a threshold chosen at -43 dB. The mean tissue signal is calculated based on the mean signal level of all datapoints below -50 dB. Datapoints with signal intensity between -50 dB and -43 dB could be caused by motion artefacts or possibly MBs that are not responding adequately to the ultrasound. Text explaining the CTR analysis has been added in line 225-233.

8- Is it possible that your inflammation model has some variability (hard to know with N=1) ? Could Rabbit 3 have less inflammation than rabbit 2 explaining the change in brightness and histology ? Could you analyse ICAM1 expression using histology ?

We agree that immunohistochemical analysis would strengthen the paper. IHC was tried, but the antibodies and methods used were not suitable for sufficient signal from ICAM on endothelial cells in rabbit. The background staining was too strong. We could therefore not conclude on the basis of IHC data.

From the ultrasound images, the size of the inflamed areas was similar for all three animals, but whether the immunological reaction and amount of available ICAM-1 for the MBs to attach to is the same is something we cannot conclude. A larger study with more animals would be needed for a more comprehensive statistical analysis. In this study our aim was to show proof-of-concept of the imaging method both in vitro and in vivo.

9- The selections of the analyzed sections in the histology analyses need to be shown. It is unclear how the 11 areas in the inflamed tissues and 7 areas in control muscle were selected.

This has been clarified in line 251-252 in the manuscript. The number of MBs in the ROI (1x1mm2) with the maximum number of MBs in each section was stored.

10- How were in vivo CTR values computed ? How did you choose MB signal vs tissue signal ? In which frame in the video ? Please describe the method and report mean, standard deviation, #MB analyzed and how they were chosen.

CTR for in vivo ultrasound images was calculated in EchoPAC, the PC software for postprocessing of ultrasound images from GE scanners. Circular ROIs with diameter of 0.5 mm was chosen and placed where there were MBs in some of the recorded frames. (see screenshot above) Since the MBs are in motion in the recordings done during or just after MB injection or due to translation of the transducer in the recording when MBs were stationary in the tissue, the MBs typically caused local peaks in the trace resulting from each ROI. The signal level from the local peaks (representing MBs) were compared with the background signal level. This explanation has been added in the Method section, see Line 225 to 233, and more detailed results are given in in Line 354-356 and 363-367. The tissue signal was found to be – 53.1 ± 2dB and contrast signal from flowing MBs were -36.3 ± 4.6 dB and signal from stationary MBs were -39.8 ±2.5 dB, resulting in CTR of 16.8 dB and 13.3 dB for flowing and stationary MBs respectively.

11- It would be interesting to stain for endothelium to confirm the compartment of the microbubbles after 48h in vivo. Are they in the vasculature ? Uptaken by leucocytes ?
We agree that it would be interesting, but unfortunately it is not possible at this timepoint.

The microbubbles are around 3 micrometer in diameter and it is not expected that they can extravasate from normal vessels and into the extracellular matrix. In the paper by Härmark et al (2015), it was concluded that the PVA microbubbles similar to the ones used in this work, were found inside the vasculature, and taken up by or in the vicinity of macrophages. This is added in the discussion on Line 540-542.

MINOR

You mention imaging during MB injection (L204) but don’t show images. Since two B-mode images are used to compute the contrast image, using one for B_mode would allow perfect coregistration and allow anatomical correspondence in Figure 7.

Imaging of inflow will be added as supplementary data. (commented in line 356)

During the in vivo experiments, the processing of the novel contrast enhances images is done on the scanner, prior to storing images (dicom), hence we do not have access to the b-mode images which form the basis of the contrast images. In the in vitro recordings we were able to store RF data, where full access to all received frames were possible. However, only up to 9 frames were available at a time, and hence we prioritized to record longer sequences of dicom format in the in vivo situation rather than just a few frames of RF data.

Please verify the excitation wavelength for your fluorophores L217-L218

The excitation wavelengths have been checked and found correct, and the emission filter data has now also been added to the text. See line 244-246.

Figure 7 : Perhaps using the first imaging pulse as the B-mode would help coregister B-mode and contrast images. Right now you are selecting an arbitrary frame from the cineloop than is not in the same position as the B-mode

See previous comment on this matter.

Figure 7E : Correct label on the right side : It should be 24h correct ?

Your observation is correct. Thanks for noticing. It is changed in the new version. In addition, it was discovered that the scale of the dynamic range on the contrast images was wrong. This has now been corrected, and the correct dynamic range for the images is 30 dB.

Figure 8 : Use arrows to indicate the different tissues (including the puss filled region and normal muscle tissue) and indicate features described in the text (inflammatory cells, dead leukocytes, cellular debris, granulocytes). Explain why these two sections were selected.

We have added squares in the HES-image which correspond to the squares in the confocal image to clarify which areas of the tissue are enhanced in the fig 8C and 8D. The area of healthy muscle is shown in a green square, whereas the inflamed region is marked with a white square.

The sections were chosen to show that there were many MBs present in an area of high inflammatory activity, whereas in the muscle area 1 mm away from the pus-filled region there are hardly any MBs detected. A comment on this has been added in the text of the manuscript, se line 433-436.

Reviewer #3: This manuscript describes a contrast pulse strategy to interlace 2 modifications pulses between 2 identical imaging pulses for the specific application of thick-shell targeted microbubbles. While such method can’t pick-up non-linear echoes like Pulse Inversion, it should be sensitive to agent motion and disruption. The ability to detect bounded microbubbles in-vivo 22 hours after injection is impressive. My main concern is that the authors use identical pulses for the imaging and modification components in this manuscript which is very close to a Doppler sequence (identical to a Doppler sequence if the PRF is constant). The in-vivo section uses 2 pulses which is identical to a (2-pulse) Doppler sequence. Doppler does work at detecting bubbles at high MI regimes but is not novel. While I understand the research limitation of the clinical system, my suggestion to the authors is to either design a simple single transducer in-vitro experiment demonstrating the advantage of the intermediate pulses (e.g. much longer pulse, different frequency, etc.) to distinguish the method from Doppler, or rephrase the manuscript as using Doppler for targeted agent which by itself could be novel. In the later case, measuring the Doppler spectrum of the targeted agents and comparing it to tissue would be relevant.

The introduction is also missing significant background on existing pulse sequences. Amplitude modulation in particular is relevant to this work as its application has been demonstrated to gas vesicle which closely resemble thick shell microbubbles. It is also routinely available on clinical ultrasound systems.

The aim of the current paper was not to describe various contrast pulse sequences and reference 3 provides a good overview of such sequences. The main point is that current non-destructive contrast sequences typically rely on the generation of a nonlinear response from the bubble that is adequately strong at mechanical indexes low enough for the wave propagation in the tissue to be essentially linear.

Gas vesicles typically have very thin protein shells (approx. 2nm or less) and the vesicle itself has a cylindrical dimension with diam of 50-100 nm and length of 100-1000 nm. The PVA bubble has a thick polymer shell of approx. 200 nm. Our GE Vivid scanner did not have an implementation of AM and there is no reason to believe that AM will work adequately for thick-shelled PVA bubbles. The buckling-effect and compression-only behavior for example seen with SonoVue (having a lipid shell of approx. 2 nm) is unlikely with a thick polymer shell of approx. 200 nm.

Comments:
(introduction) ‘Thick-shelled MBs can have improved circulation half-lives, incorporate larger amounts of drugs for enhanced drug delivery or facilitate targeting for use in molecular ultrasound imaging. However, methods for robust imaging of thick-shelled MBs are currently not available.” Comment: What does the author consider thick shell? Would Sonazoid count as a stiff shell (compared to Sonovue/Definity)?

We consider MBs with shells thicker than 100 nm as thick-shelled MBs. This is specified in the introduction. (line 37)

(introduction) The introduction does not provide information about the proposed novel imaging technique. Also, the background on general pulse sequence literature is missing from the introduction.

The purpose of the paper is not to give an extensive overview of all other methods, but we have referred to literature and described briefly their dependence on low MI for nonlinear detection of MB without invoking too much nonlinear backscattering from surrounding tissue. See lines 26-50.

Two more references have also been added:

4.	Phillips, P. *Contrast pulse sequences (CPS): imaging nonlinear microbubbles*. in *Ultrasonics Symposium, 2001 IEEE*. 2001. IEEE.

5.	Eckersley, R.J., C.T. Chin, and P.N. Burns, *Optimising phase and amplitude modulation schemes for imaging microbubble contrast agents at low acoustic power.* Ultrasound Med. Biol., 2005. **31**(2): p. 213-219.

(p.5) ‘MBs used in the current experiments were not well imaged by conventional harmonic imaging schemes based on pulse inversion’ Comment: What about amplitude modulation (AM)? In particular, one would suspect that AM would perform well for thick shell agents due to their buckling dynamics (see Marmottant model)

See previous comment.

(p.6) The description of the pulse sequence comes really late in the manuscript and is hidden with other experimental details. I would move it closer to the introduction.

The method description in the introduction is expanded to (line 46-50):

*We propose a novel multi-pulse imaging method where a combination of imaging pulses are combined with intermediate manipulation pulses at high mechanical index. Radiation force both from the imaging and manipulation pulses, in addition to possible nonlinear effects from changes in the shell result in contrast enhancement of thick-shelled MBs with adequate tissue suppression.*

And a thorough description of the method is included in the methods chapter under “In vitro ultrasound imaging”.

(p.6) ‘first and the last pulse were identical, and were used for imaging, whereas one or several intermediate pulses could be included for additional manipulation of the MB’. Comment: If the first and the last pulses are identical, then the pulse sequence is not extracting any nonlinear echo information but instead will pick-up either motion, bubble disruption or partial bubble disruption. Furthermore, here, both the imaging and intermediate pulses are identical which means the authors are simply using a Doppler sequence (in particular if the PRF is constant). This would need clarification in the manuscript.

It is true that the pulse sequence does not primarily rely on nonlinear bubbles response (even if nonlinearities due to alterations in the shell can enhance the detection). The main effect is thought to be a physical translation due to ultrasound radiation force from the incident pulse on the bubble. One might argue that the technique has similarities with conventional Doppler but Doppler techniques are made for imaging an existing flow, not to induce a translation due to ultrasound radiation force as with our method. That is why we want to use imaging pulses tailored to the organ size and depth of interest and intermediate manipulation pulses that are tailored to maximize the radiation force on the PVA bubbles. Also, Doppler makes use of transmit pulses consisting of several pulse oscillations (resulting in reduced range resolution) and several pulse packets needs to be transmitted in each beam direction for spectral analysis (to construct an adequate clutter filter for suppressing strong, slow moving scatterers and to estimate blood velocities).

(p.7 l.137) ‘and acquisition schemes with MI of 0.7 and 1.0’. Comment: While I expect hard shell bubbles to be more resilient to pressure, those are very high MI when compared to conventional contrast imaging. For traditional non-destructive imaging the norm is MI~0.06-0.1. The introduction made is sound like the contrast sequence needs to be non-destructive. This should be clarified in the manuscript.

As stated in line 359, we see no clear signs of destruction, so we consider the method to not rely on destruction of thick-shelled MBs. If the same MI was used on thin-shelled MBs destruction would be expected.

(p.7) ‘The ultrasound transducer was fixed with a clamp and MBs were imaged both during low speed flow and when the pump had stopped and the MBs were stationary.’ Comment: Were results computed for each condition or combined? Flow introduce motion between pulses and yield a significant increase in the CTR which may shadow other effects from the intermediate pulses.

The contrast to tissue ratio for the in vitro experiments was computed from images which were recorded after the flow pump had stopped, hence there is no bubble motion due to flow to influence the CTR. A comment has been added to clarify, see line 161-162.

(p.10) ‘A frequency of 9.5 MHz and MI of 1 was used in the contrast images, and 12 MHz and MI of 1 was used to acquire the B-mode images. In order to minimize tissue signal and increase the framerate, the in vivo recordings were done without any intermediate pulses, hence only the two imaging pulses were transmitted along each beam line. Comment: In this case, the contrast sequence is simply a 2 pulse Doppler sequence. Doppler sequence do work at imaging bubbles, but only if they are moving are destroyed by the imaging pulse. Doppler was actually the original detection scheme for microbubbles (before PI and AM were invented), and has been documented in literature. I recommend that the authors discuss this in the introduction.

See previous comment on the comparison to Doppler imaging above.

(p.12) ‘When comparing the amount of MBs in the image in Figs 5A and 5B, it is apparent that not all MBs become visible with this method’. Comment: Could this indicate that the high MI has detrimental effect on the thick shell microbubbles or that microbubble coalesce into larger bubbles that become easier to detect?

See also previous comment regarding bubble response from numerous pulses.

It is not likely that they coalesce into larger bubbles, since their shell is a thick polymer, and not very reactive. It is more likely that not all bubbles are equally affected by the US pulses, and hence does not provide any difference in the signal between the two imaging pulses. Longer intermediate pulses or even higher pressure could help, however that was not possible to implement on the clinical scanner used in the proof-of-concept study. This is commented in lines 492-493.

(p.13) ‘The backscattered signals from 6 to 10 MBs in each image were analyzed, and the detected mean delay between the first and last imaging pulse along the scanline through the MB and the mean CTR are presented in Table 1.’ Comment: This CTR algorithm seems biased. Bubbles are supposed to be everywhere in the flow channel; a more robust metric would be to compare larger ROI in the tube and tissue.

This method is chosen because a low concentration of MBs is used in order to show that single MBs can be imaged. If a large area of the flow tube is chosen, a much higher concentration of MB must be used in the flow, otherwise the dark pixels between MBs will affect the CTR negatively, and not be representative of the actual CTR of the technique.

(p.14) ‘The CTR of circulating MBs immediate after injection was 15-20 dB.’ Comment: It would be relevant to create a new figure with the Contrast image a several time stamps after the injection in order to justify the 15-20dB claim.
(Discussion) It should be discussed why the authors didn’t consider Amplitude modulation, which is the most commonly used contrast pulse sequence on clinical systems. It is also worth noting that gas vesicles (nanobubbles with thick shells) were successfully imaged with AM due to their buckling dynamics [reference: Acoustic Behavior of Halobacterium salinarum Gas Vesicles in the High-Frequency Range: Experiments and Modeling, E. Cherin et al., UMB, Vol 43, Issue 5, pp.1016-1030] and is therefore a promising candidate for their thick shell microbubbles.

See previous comment on in vivo CTR analysis. In addition, a video of MB injection in Rabbit 3 has been added as supplementary data, and the Q-analysis data exported from EchoPAC is also included as supplementary data.

Gas vesicles typically have very thin protein shells of approx. 2nm.

(p.19 l.428) ‘Tissue motion may especially be a problem in the abdominal and thoracic areas, where breathing and the beating heart may cause strong artefacts if no motion compensation is included in the implementation. In the current work, a relatively stationary organ was imaged (the thigh).’ Comment: This is precisely the same limitation of Doppler imaging. Doppler mitigates this issue by using a wall-filter which rejects low Doppler frequencies. I recommend to measure the Doppler spectrum from these microbubbles and compare it to the in-vivo Doppler spectrum of tissue. One would expect the microbubble spectrum bandwidth to broaden compared to tissue Doppler due to signal decorrelation.

Analyzing spectra is a nice idea, however it is not possible from the data we have collected here. Raw data from in-vivo imaging was not recorded, hence it is not possible to analyze spectra in retrospect.

The suggested imaging method is perhaps best suited for imaging of essentially stationary organs or for triggered imaging of less stationary organs. For the construction of a wall or tissue filter, numerous pulse packets must be applied in each transmit direction. This is time-consuming and the tissue will have more time to move and decorrelate. With our method, the result is not so much a constant or continuous bubble velocity (as is the case for blood flow imaging or tissue Doppler imaging). Constructing a wall-filter for suppressing tissue and trying to induce a bubble velocity that is above some threshold is perhaps not feasible.

(p.19 l.437) ‘We have not been able to verify the presence of such effects in the current work’. Comment: That being said, it is unlikely that the acoustic pulses moved the bounded bubbles in the in-vivo experiment.

We agree. Knowing exactly what the cause of the detection signal from each MB in an in-vivo situation is impossible (except for maybe in intra-vital microscopy at very high magnification and frame rate). In vitro, it is possible to study single bubble behavior, as has been done in Kohtapalli at al (2015).

Editorial
(p.5 l.87) ‘[…] has been described earlier’ Comment: Please rephrase as ‘has been described by Cavalieri et al […]’

Thanks for noticing. This has been corrected.

6. PLOS authors have the option to publish the peer review history of their article ([what does this mean?](https://eur03.safelinks.protection.outlook.com/?url=https%3A%2F%2Fjournals.plos.org%2Fplosone%2Fs%2Feditorial-and-peer-review-process%23loc-peer-review-history&data=04%7C01%7Csigrid.berg%40sintef.no%7Cbc069de421ab4e71b8d708d9e6718737%7Ce1f00f39604145b0b309e0210d8b32af%7C1%7C0%7C637794198994625074%7CUnknown%7CTWFpbGZsb3d8eyJWIjoiMC4wLjAwMDAiLCJQIjoiV2luMzIiLCJBTiI6Ik1haWwiLCJXVCI6Mn0%3D%7C3000&sdata=U6njwwX2FwcawUc%2Byn1N9MnXlzaU7oYMDvYdpMqB4XI%3D&reserved=0)). If published, this will include your full peer review and any attached files.


If you choose “no”, your identity will remain anonymous but your review may still be made public.


**Do you want your identity to be public for this peer review?** For information about this choice, including consent withdrawal, please see our [Privacy Policy](https://eur03.safelinks.protection.outlook.com/?url=https%3A%2F%2Fwww.plos.org%2Fprivacy-policy&data=04%7C01%7Csigrid.berg%40sintef.no%7Cbc069de421ab4e71b8d708d9e6718737%7Ce1f00f39604145b0b309e0210d8b32af%7C1%7C0%7C637794198994625074%7CUnknown%7CTWFpbGZsb3d8eyJWIjoiMC4wLjAwMDAiLCJQIjoiV2luMzIiLCJBTiI6Ik1haWwiLCJXVCI6Mn0%3D%7C3000&sdata=7zVxG22c7pcTa0cos48Gs72N0PtndeEel0fF6YVwv2M%3D&reserved=0).

Reviewer #1: No

Reviewer #2: No

Reviewer #3: No
